# The Treatment of Contracted Bladder by Hot Water Dilatation

**Published:** 1890-01

**Authors:** I. S. Stone

**Affiliations:** Lincoln, Va.


VOL. VI. JANUARY, 1890. No. 11.

### Original Communications.

### THE TREATMENT OF CONTRACTED BLADDER BY HOT WATER DILATATION.

BY I. S. STONE, M. D., LINCOLN, VA.

During the past few years certain protracted cases of cystitis, occurring chiefly in women, have been observed by the writer, which have resisted all known forms of medical treatment, and necessitated some surgical or mechanical measure of relief.

The chief difficulty in the way of successful treatment heretofore would appear to be due to a want of comprehension of the result of long continued disease of this viscus. Another difficulty presents itself, in that surgeons have not learned to carefully ascertain the exact size, i. e., the capacity of the bladder. For instance, we are directed in the text-books to use a sound carefully to measure the depth of the bladder. If fifteen c. m. (six inches) (Baker), it is supposed not to be smaller than normal. Such an examination may lead to error, as a bladder may be fifteen c. m. in depth and yet contain very little fluid. I have seen several cases in which the sound would enter but two to three inches, and yet by proper measurement with water they would hold more than this depth would indicate. We usually resort to various expedients to definitely learn the shape, size, capacity, etc., of other organs; would it not be well to apply the same methods to the organ in question, the bladder? The sound is valuable as an aid to diagnosis; the endoscope, with proper illumination, has almost revolutionized the treatment of intra-vesical diseases, but it remains for surgeons generally to measure carefully the capacity of the bladder in any case of long continued disease thereof.

Kussmaul, in 1869, called attention to the treatment of certain diseases of the stomach by lavage. This involved accurate measurements, although no mention is made of it save to show when over-distended. Freich distended the stomach with gas, but only to outline the organ. I respectfully urge the employment of hot water, plain or medicated, with boric acid to ascertain the size of diseased bladders, and to dilate them if found less than normal size. The patient rarely gives a correct statement as to the quantity of urine each day, or at each act of micturition. Such statements are about as reliable as their representations as to color, appearance, sediment, etc.; therefore it would appear unnecessary to urge at great length the importance of the above remark.

Let me ask that it be taken for granted that the pathology of cystitis is well understood, so far as its effect upon the three principal divisions of tissue in the bladder is concerned. That the muscular coat, when continued in extreme contraction (eccentric or concentric), becomes permanently fixed in this state, simulating at first a tonic spasm.

That any fluid, healthy urine not excepted, but especially urine of high specific gravity, or loaded with pus, or swarming with bacteria, and strong with ammonia, must arouse the various coats of the inflamed bladder to exert themselves in an agony of contraction, which in weeks, or in months, becomes permanently fixed.

*Causation.*—My opinion is decidedly favorable to the idea that cystitis is always secondary to other disease beyond or outside the bladder. Exception is made in case of traumatism, or from active poisons.

I reject emphatically the possibility of cystitis from exposure to cold, and very much doubt the possibility of idiopathic cystitis. Later writers generally favor this opinion, notably Thompson, Baker, Harrison *et al.* Thus we have causes referable to the kidney or ureters. (The writer has had two cases from tubercular kidney); the various diseases of the peritoneum adjacent to and covering the bladder, epicystitis, etc.

The exceeding hypertrophy of the walls of the bladder is often quite astonishing, and when the patient is examined per rectum, or vaginam, the sensation is like that given to the finger by a fibroid of the uterus. It is in vain that we may expect this great thickening to disappear in any case by drainage, internal medication or irrigation. It is plausible to mention pressure, if pressure could be exercised. I suggest that by means of hot water distention such a bladder may be treated, dilated and exercised. It is to this form of treatment, aided by the injection of iodoform, that I now wish to call your attention.

*Treatment.*—The failure of irrigation, as a method of treatment in cases of contracted bladder from cystitis or other cause, would now appear to be due in part to the impossibility of thus cleansing such an irregular surface. We know there are many pockets and perhaps duplications in such bladders, not to speak of the shaggy coat shown by means of the endoscope ; besides many glands, probably with septic contents. Hence a thorough dilatation would appear imperatively necessary, if a diseased bladder be actually cleansed. I may say in passing that Dr. T. A. Emmet does not favor irrigation. Neither does Agnew, who says in his Surgery (Vol. 11, p. 530): "The introduction of liquids, plain or medicated, into the bladder, either as washings, or by irrigation, is of very doubtful propriety. Convinced of their inutility, and of their bad effects, I have for some time abandoned their use.
* * * *"

I will not allude in this paper to dilatation of the urethra or to drainage by catheter. The former applies only to the urethra, or its vicinity ; the latter cannot cure a contracted bladder.

According to Dr. Weir, of New York, in thirty-six cases requiring cystotomy, eleven died, seventeen were cured or nearly so, four were slightly relieved, four not benefited.

Therefore the operation of Dr. Emmet would appear not to be without danger, or at least it is far from always proving successful. It needs but to be tried, to know how exceedingly distressing the condition of the patient becomes, from the flow of urine through the vagina.

Skene has invented an instrument, however, which may possibly overcome this diffculty in some cases. Moreover Emmet's operation of cystotomy is worthy of a position unattained by any other method of treatment, for it may give great relief where nothing else can succeed, even should it fail in curing the contraction.

In the treatment of contracted bladder by dilatation, it must be taken for granted that the case is a suitable one to receive the proper distention. It would, of course, be bad surgery, to commence dilating an organ already attacked by malignant disease, or so far weakened by any pathological condition as to render such a procedure hazardous. We will take it for granted that the organ is contracted as a result of long-continued irritability, or from cystitis from any cause, either as a result of pyelitis, or other disease of the kidney, or from other causes in the pelvic cavity, outside the bladder, such as pericystitis, or finally from infection, as by foul catheters.

The patient is given morphia sulph. gr. ¼, atropia sulph. gr. $\frac{1}{200}$ hypodermatically. She is placed on her back on a table for convenience, although it would answer to arrange the bed with the patient thereon to suit the operation. A soft catheter is at once inserted into the bladder, and often the urine has escaped, hot water, temperature 110, is thrown into the bladder, until the patient will no longer bear it. This is allowed to escape, and is measured,

NOTE.—Dr. Emmet reports in his Gynecology ten of sixteen cases cured by cystotomy.

giving the full size of the bladder in its present condition. As the morphia gradually becomes absorbed, the patient will bear still further distention; each time perhaps one drachm may be added to the capacity of the bladder. I prefer using a rubber ball syringe, holding two to four ounces. The pressure of the hand is safer than that of the tube and funnel or any instrumental gauge, as the patient is generally unable to resist the tendency to strain, owing to the tenesmus produced by the expansion of the bladder. As each seance should continue thirty to sixty minutes, the bladder may be filled and emptied many times, and at first the operator must be well satisfied if the gain is only one or two drachms in a bladder whose capacity is perhaps only two ounces. As the patient becomes fully under the influence of the morphia, the water may be increased in temperature to 120° or 125° F. The very best effect follows its use when at this temperature.

In concluding the seance, if the case has never had a trial of nitrate of silver, and there is surely ulceration present, the drug may be tried gr. x to xx or xxx ad. ℥j iaqua dest. Enough of the solution should be thrown in to at least distend the bladder to one-half its capacity, but never to the full extent, as the drug is not intended for the freshly separated portions, but only for the ulcerated surface. I do not personally approve of silver nitrate, either in the uterus or bladder, under any circumstances, but mention the above on the authority of others. I am fully convinced that if used without the hot water method of treatment here proposed, still greater contraction must result, especially if the stronger solutions are tried, as by some recommended. I very much prefer iodoform, made with gum acacia into a mucilaginous mixture; ten grains may thus be thrown into the bladder in an appropriate quantity of liquid, so that the patient will allow it to remain for some hours if possible. The bladder will thus be coated with iodoform for several days before the urine can dislodge it. I have never seen any evidence of its absorption into the circulation, or witnessed any but good results from this method of using it. Cocaine has not answered well even in ten per cent. solution to relieve pain inside the bladder. It will not prevent pain or allay it, in the treatment, or that following the nitrate of silver.

It has always proven a failure except for some slight operation on the urethra, where it appears to act well. My practice has been to follow up the distention with hot water, the patient under the influence of morphia—by daily distention without the anodyne. An effort should be made to keep the bladder open to the point already reached, until the period of five days has passed, when the patient will be ready for the morphia and still further distention. It will be found that on the first and second days after the morphia and full distention, the patient will not be willing to take the full amount of water, even after several trials, occupying the space of an hour, but after this the full quantity will be allowed. It will be ascertained on trial that water at a temperature of 110 to 125 Fahr. will give far better results than at a lower degree. One word in regard to the possibility of hemorrhage. I have rarely succeeded in accomplishing much in these cases, without causing a slight hemorrhage. It at first caused me some anxiety, and I remember writing to Dr. Gross about it. He advised me never to carry the dilatation to that extent. Experience with the method, however, served to show that a silght hemorrhage did no harm, and in fact I have never seen any bad results. The oozing always ceased promptly when the bladder was allowed to contract. I have frequently seen one or two drachms (estimated) at one seance, the water returning highly colored. I fancy many doubts will arise as to the safety of this method. I have thus far found only one reference to rupture of the bladder caused by dilatation. Skene (Diseases of Women, p. 752). The patient had long worn a hard catheter fastened in the bladder, which, by pressure, had perforated two of the coats of the bladder, leaving only the peritoneal coat to rupture when the effort of dilatation was made. The autopsy clearly demonstrated this as the cause of the accident. As to the amount of distention authorities differ. Some would suggest not to the full extent, that it is dangerous to exceed eight ounces if the bladder has been reduced to one or two ounces. My experience is, that a bladder will again contract, unless it is brought out full and free from internal adhesions, to eighteen or twenty ounces. Even then it requires occasional stretching and care to prevent contraction from recurring. If hemorrhage has frequently occurred when any important gain in size has been made, it will probably cease when the full size, sixteen to twenty ounces, has been reached, and a gain in capacity of one to two ounces may be made at one sitting, showing plainly that the bladder is folded upon itself, as it contracts from disease. This may be easily understood by referring to Skene's valuable diagrams, pp. 773 and 774, Diseases of Women.

The following cases are very briefly told for illustration:

Case I.—Male, age thirty-eight, had chronic cystitis for three years, which resulted in contracted bladder, without the knowledge of his physician, who had faithfully continued the drugs supposed to influence the bladder. When first seen (December, 1885) there was evidence of pyelitis or abscess of the right kidney. This proved to be an abscess, which was drained through the usual lumbar incision, used in nephrotomy. The patient, although greatly relieved, gradually sank in a few weeks from general tuberculosis. So far as the bladder is concerned an effort was made to dilate it, which failed because of the very reduced condition of the patient. Its capacity was less than an ounce, and at the autopsy appeared as a mere pouch at the terminations of the ureters.

Case II.—Miss M., age twenty-five, had been treated for cystitis for two or more years, with apparent success at first, but afterwards all treatment failed, and, when I first saw her in 1886, her bladder would hold less than four ounces full distention. As I suspected renal disease, she, at my request, consulted the late Dr. S. W. Gross, of Philadelphia, who had her in charge for nearly two months. While under this eminent surgeon's care, the bladder was increased in capacity from two drachms to two ounces. She came under my care at once after this, and for a time the irrigation and injections of silver nitrate were continued.

Note.—Case No. 2 is reported in Journal American Medical Association for November, 1889.

But owing to the slow progress of the case, and stimulated by the resolution formed while treating Case 1, I tried forced distention with hot water. The surgeon who had previously had charge of the case declined to admit the possibility of renal involvent, which at the autopsy, two years after she came under treatment, was proven.

The urine was heavily laden with pus and epithelium, which greatly diminished as the bladder was thoroughly dilated to nineteen ounces capacity. This patient almost entirely recovered from the cystitis and contracted bladder, but succumbed to the turbercular disease of her right kidney.

CASE III.—Miss——, aged thirty-six, had frequent micturition and supposed cystitis for three years before coming under my observation, although she had not become an invalid in appearance. I could not discover much evidence of cystitis, save the frequency of urination and dysuria. She was obliged to rise three or four times each night, and never could retain her urine longer than three hours. At the first examination I discovered an anteflexed uterus, and quite naturally thought this the cause of the cystitis, which had in turn caused the contracted bladder, which would not retain over three ounces without acute pain and uncontrollable desire to pass urine. As all previous treatment had failed to relieve this patient, I at once commenced to dilate her bladder and in three months she could retain her urine for eight hours and slept all night without rising. She had abundant courage and assisted greatly in the treatment, by retaining her urine as long as possible. After learning that I had to do with what may be called pericystitis, that the uterus was drawn over upon the bladder and tied there by adhesions, the difficulty was readily solved. This lady has apparently recovered, although I could not learn that she had the symptoms of cystitis primarily. It would appear that contracted bladder may easily result from adhesions from pelvic inflammation, as it may result from direct inoculation, as with an infected catheter.

CASE IV.—Male, with large prostrate, aged seventy-five. This patient had tried all forms of treatment, such as by sounds, and by wearing catheters, and even the continued use of the galvanic current. His bladder would retain three ounces with forced distention. Could not take morphia. Treatment cured the cystitis and increased his capacity for urine from three to six ounces. He is greatly relieved and rarely uses a catheter.

---

NOTE.—In reference to the priority of this method of treating contracted bladder, I was unaware that other writers had claimed any degree of success. A careful search of the Index Catalogue of the library of the Surgeon General's office at Washington gave no evidence of any efficient trial of this method.

During the preparation of this paper, the American Journal of Obstetrics, for September, came with Dr. Sims' interesting paper, read March 5th, before the Obstetrical Society of New York. I find, however, that Dr. Sims' paper distinctly refers to contracted bladder in young women who may have incontinence, etc., but he does not claim it as valuable in all forms of contracted bladder, including that of cystitis. A full report of Case 2 was prepared in June for a medical journal, but was, owing to accident, not printed. Therefore I confess to being antedated by Dr. Sims, whose first case occurred eight years ago, while mine occurred in 1885, or four years since.

The discussion which followed Dr. Sims' paper shows that in Sweden one Dr. Oscar Nissen, of Christiania, uses this method with great success. (See American Journal Obstetrics, p. 961, September.)

I can heartily commend Dr. Sims' article, and recognize how true his remarks are in reference to the amount of perseverance required on the part of the patient, and I may say on the part of the surgeon as well.

